# Chronic Pain and Comorbid Emotional Disorders: Neural Circuitry and Neuroimmunity Pathways

**DOI:** 10.3390/ijms26020436

**Published:** 2025-01-07

**Authors:** Meihong Li, Kepeng She, Pengfei Zhu, Zhen Li, Jieqiong Liu, Fang Luo, Yingze Ye

**Affiliations:** Department of Anesthesiology and Pain Medicine, Hubei Key Laboratory of Geriatric Anesthesia and Perioperative Brain Health, Wuhan Clinical Research Center for Geriatric Anesthesia, Tongji Hospital, Tongji Medical College, Huazhong University of Science and Technology, Wuhan 430030, China; lmh18236985650@163.com (M.L.); skepeng@163.com (K.S.); zpf_wx@outlook.com (P.Z.); 15807197038@163.com (Z.L.); wendao.shuiji@163.com (J.L.)

**Keywords:** pain comorbid emotional disorders, neurotransmitters, neural circuits, inflammation, immune response, therapeutic strategies

## Abstract

Chronic pain is a multidimensional experience that not only involves persistent nociception but is also frequently accompanied by significant emotional disorders, such as anxiety and depression, which complicate its management and amplify its impact. This review provides an in-depth exploration of the neurobiological mechanisms underlying the comorbidity of chronic pain and emotional disturbances. Key areas of focus include the dysregulation of major neurotransmitter systems (serotonin, gamma-aminobutyric acid, and glutamate) and the resulting functional remodeling of critical neural circuits implicated in pain processing, emotional regulation, and reward. Given the contribution of neuroimmune mechanisms to pain chronicity and mood disorders, we further conducted an in-depth investigation into the role of neuroimmune factors, including resident immune cells, infiltrating immune cells, and the release of inflammatory mediators. This review further discusses current therapeutic strategies, encompassing pharmacological interventions, neuromodulation, and integrative approaches, and emphasizes the necessity of targeted treatments that address both pain and emotional components. Finally, it identifies gaps in the current understanding and outlines future research directions aimed at elucidating the complex interplay between chronic pain and emotional disorders, thereby laying the foundation for more effective and holistic treatment paradigms.

## 1. Introduction

Pain is an unpleasant sensory and emotional experience associated with actual or potential tissue damage. It is a fundamental component of normal sensory perception and is widely recognized as the fifth vital sign in human health [1]. However, chronic pain refers to pain that persists or recurs beyond the expected time for tissue healing, typically defined as pain lasting 3 months or longer [2]. Studies have shown that prolonged or severe chronic pain can lead to psychological issues, including low mood, anxiety, and depression [3,4,5]. Depression is an emotional disorder characterized by a persistent sense of sadness and/or an inability to experience pleasure, often accompanied by impaired daily functioning, such as weight loss, fatigue, sleep disturbances, and pain [6]. Epidemiological studies indicate that approximately 20% to 90% of patients with chronic pain are diagnosed with depression, and the lifetime prevalence of depression among individuals with chronic pain is more than three times higher than in those without chronic pain [7]. Bair et al. reported that the average prevalence of major depression among chronic pain patients in pain management settings was 52%, while 65% of patients with depression in primary care settings also experienced comorbid pain [7].

Despite substantial clinical evidence showing a strong comorbidity between pain and depression, the underlying neurobiological mechanisms are challenging to study in patients. Borsook et al. [8] proposed the Combined Reward Deficiency Antireward Model to explain the pathophysiology of chronic pain. This model proposes that acute pain triggers the brain’s reward system, which helps to alleviate pain. However, if pain persists and fails to resolve, it suppresses the reward and motivation centers in the brain, diminishing the motivational salience of natural reinforcers—a state known as reward deficiency. To compensate for this reward deficiency, the antireward system releases stress-related chemicals, which result in reduced dopaminergic activity and changes in both pain perception and associated comorbidities. These changes, referred to as the antireward state, include reductions in dopamine receptors, decreased dopamine synthesis, and increased dopamine transporter levels. The pathological state of chronic pain exacerbates both pain perception and emotional comorbidities, such as addiction, depression, and anxiety. To investigate the neurobiological mechanisms underlying emotional disorders induced by chronic pain and explore potential interventions, researchers have increasingly turned to imaging studies and preclinical animal models. Examples include inflammatory pain induced by Complete Freund’s Adjuvant (CFA) [9], neuropathic pain induced by spared nerve injury (SNI) [10], and visceral pain induced by colitis [11]. These models provide crucial insights into the neurobiology of chronic pain and its comorbid emotional disorders, offering potential avenues for therapeutic strategies.

The objective of this review is to summarize and analyze recent advances in the neurobiological research on emotional disorders comorbid with pain. It examines the dysfunction of major neurotransmitter systems, such as serotonin (5-HT), gamma-aminobutyric acid (GABA), and glutamate, as well as disruptions in key neural circuits, including pain perception pathways, emotion regulation circuits, and the reward system. Additionally, the role of inflammatory and immune mechanisms in the development of emotional disorders induced by chronic pain is discussed. Finally, this review evaluates existing therapeutic strategies and proposes future research directions to more effectively address this complex issue.

## 2. Neurobiological Mechanisms of Pain-Induced Emotional Disorders

### 2.1. Neurotransmitter Dysfunction

The relationship between chronic pain and mood disorders is complex, and there is an extensive interaction at the neurobiological level. In the comorbidity of chronic pain and mood disorders, changes in several key neurotransmitters are considered the basis of their interaction. In Table 1, we provide an overview of the sources and roles of monoamines, the inhibitory neurotransmitter GABA, the excitatory neurotransmitter glutamate, and corticotropin-releasing hormone (CRH) and oxytocin in the comorbid condition.

#### 2.1.1. Monoaminergic Neurotransmitters

The imbalance of monoaminergic neurotransmitters, such as 5-HT, norepinephrine (NE), and dopamine (DA), is critically implicated in the onset of depression [12,29]. The 5-HT system primarily influences sleep and sexual behavior. NE pathways are closely associated with depressive symptoms, such as apathy, loss of interest, disrupted sleep patterns, fatigue, and cognitive impairments. DA, on the other hand, is involved in the reward system and motivation [12]. In healthy individuals, the interplay among serotonin, dopamine, and norepinephrine functions in a coordinated manner to regulate emotions, pain perception, and stress response. However, in individuals with comorbid pain and depression, the dysregulation of these three neurotransmitters creates a vicious cycle [30,31].

The descending serotonergic and noradrenergic pathways are considered modulators of pain perception, functioning through the activation of ‘on’ and ‘off’ cells. The descending pain pathways are mediated by serotonergic neurons that originate in the raphe nuclei of the rostral ventromedial medulla (RVM) and noradrenergic neurons from the locus coeruleus in the dorsolateral pontine tegmentum (DLPT). These pathways converge on the dorsal horn of the spinal cord, where they play a key role in inhibiting pain signals [13,14]. Serotonin signaling in the brainstem and spinal cord is impaired, leading to a weakened inhibitory effect on pain and a reduced capacity to regulate negative emotions, thereby contributing to a vicious cycle of pain and depression [32]. Norepinephrine release from the locus coeruleus is diminished, impairing the function of descending pain modulation pathways and amplifying pain signals. Additionally, low levels of NE reduce the ability to cope with emotional stress, further exacerbating pain and depressive symptoms [32]. In addition, both chronic pain and depression are associated with disruptions in dopamine pathways, particularly in the nucleus accumbens and prefrontal cortex, resulting in diminished reward responsiveness and heightened pain perception, which becomes increasingly intolerable [8]. Moreover, early-life exposure to selective serotonin reuptake inhibitors can disrupt the maturation of circuits between the prefrontal cortex and the amygdala, resulting in not only long-term alterations in pain sensitivity but also emotional disorders such as anxiety and major depression [33]. Although current research has established the connection between monoaminergic neurotransmitters and pain-comorbid emotional disorders, pharmacological treatments targeting these systems have shown limited efficacy in alleviating such conditions. This suggests that our understanding of the precise mechanisms underlying this comorbidity remains incomplete and requires further investigation.

#### 2.1.2. Gamma-Aminobutyric Acid

GABA is the most abundant inhibitory neurotransmitter in the central nervous system, primarily mediating the inhibition of neuronal activity through GABA_A and GABA_B receptors. The amygdala consists of multiple nuclei, including the lateral nucleus (LA), the basal nucleus, and the central nucleus (CeA), and plays a critical role in the regulation of emotion, learning, and memory. In the CeA, GABAergic interneurons account for more than 95% of the neuronal population and serve as key regulators of neural circuits within the amygdala. By suppressing excessive neural activity, these GABAergic interneurons maintain the normal functioning of the amygdala, thereby playing an essential role in the modulation of emotional responses and behavioral control [18].

Ding et al. discovered that in SNI-induced chronic pain and depression models, there was an upregulation of DNA methyltransferase (DNMT) expression in the CeA of rats. The role of DNMT1 was associated with the methylation of the CpG-rich Gad1 promoter and the downregulation of GAD67, resulting in decreased activity of GABAergic neurons. The activation of GABAergic neurons in the CeA through optogenetics ameliorated depressive-like behaviors in SNI mice. Furthermore, inhibiting these neurons using optogenetic or chemogenetic approaches reversed the behavioral improvements associated with DNMT1 knockout in the same model [34]. Clinical research has revealed that patients with major depressive disorder exhibit decreased GABA concentrations in the central nervous system [19]. Similarly, Legarreta conducted magnetic resonance imaging studies and found that in veterans, emotional disorders induced by chronic pain were associated with an imbalance in the Glu/GABA ratio in the anterior cingulate cortex (ACC). The reduction in GABA in the ACC may contribute to the exacerbation of pain in these veterans [35]. Additionally, Ellerbrock provided genetic evidence linking 5-HT genes and gene interactions with emotional responses, demonstrating an association between the serotonergic system and thalamic GABA concentrations. It was inferred that individuals with heightened serotonergic signaling exhibited the highest GABA concentrations, potentially suggesting that 5-HT enhances GABAergic inhibitory effects [36]. Therefore, future research should move away from the oversimplified concept of reduced GABA neurotransmission and more accurately explore the roles of inhibitory neurotransmitters in this context.

#### 2.1.3. Glutamate

As the primary excitatory neurotransmitter, glutamate plays a critical role in the neural networks involved in pain perception and emotional regulation, primarily through its receptors. Glutamate receptor subtypes include ionotropic receptors such as NMDA, AMPA, and kainate (KA), as well as eight metabotropic G protein-coupled receptors [37]. Goffer et al. found that chronic pain selectively increases the level of the A1 subunit of AMPA-type glutamate receptors in the nucleus accumbens (NAc), leading to the formation of calcium-permeable AMPA receptors (CPARs). Pharmacological blockade of these CPARs in the NAc was shown to enhance pain-related depressive behaviors [20]. Conversely, in depression models using rats and mice, blocking NMDA-dependent firing activity in the lateral habenula (LHb) of the antireward center, thereby relieving inhibition on downstream monoaminergic reward centers, exhibited rapid antidepressant effects [21]. Mitani et al. observed significantly elevated levels of plasma glutamate, glutamine, and glycine in patients with depression during clinical studies [38]. Glutamate is a key neurotransmitter involved in pain modulation and progression, contributing to central sensitization through the activation of spinal cord neurons via AMPA and NMDA receptors. In mice with neuropathic pain-induced depression, the density of synaptic proteins and amino acid levels of glutamatergic neurons in the prefrontal cortex (PFC) were significantly increased [22]. Therefore, glutamate and its receptors play an important role in the mechanisms underlying pain and depression. The development of drugs targeting glutamate and its receptors holds potential therapeutic significance.

#### 2.1.4. Corticotropin-Releasing Hormone

The corticotropin-releasing hormone (CRH) is a peptide composed of 41 amino acids, primarily released by the paraventricular nucleus (PVN) of the hypothalamus, and it regulates various behavioral stress responses [23,24]. CRH and its receptors are located in many areas of the brain outside the hypothalamus, including parts of the limbic system, as well as in the brainstem and spinal cord’s central arousal sympathetic system (LC/NE) [25]. Research has shown that CRH expression in the PVN increases in the context of chronic visceral pain [39]. CRH neurons serve as the driving force behind the hypothalamic–pituitary–adrenal (HPA) axis stress response, playing a crucial role in the stress hypothesis of anxiety and depression. The downregulation of calcium activity in CRH neurons in the bed nucleus of the stria terminalis can alleviate anxiety-like behaviors induced by chronic stress [40]. In the trigeminal neuropathic pain model, the excitability of CRH-expressing GABAergic neurons in the mPFC significantly increased two weeks after the surgical procedure. Inhibiting this group of CRH neurons or pharmacologically blocking their receptor (CRHR1), markedly enhanced the excitability of layer V mPFC neurons and improved anxiety and depressive-like behaviors in chronic pain mice [26]. Fibromyalgia is a syndrome characterized by chronic pain and is commonly associated with depression. In patients with fibromyalgia syndrome, there is stress activation of hypothalamic CRH neurons, and the activation of the HPA axis may indirectly reflect increased serotonergic tension in the central nervous system [27]. In addition, compared to healthy individuals, chronic back pain patients showed elevated cortisol levels. These increased cortisol levels were linked to smaller hippocampal volumes and heightened pain-induced activity in the anterior hippocampus, highlighting how prolonged endocrine stress responses may alter hippocampal complex function [41]. In conclusion, developing drugs that specifically target CRH and its receptors is of significant importance for treating emotional disorders induced by chronic pain.

#### 2.1.5. Oxytocin

Oxytocin is a classic neurohypophysial hormone primarily synthesized and released into the bloodstream by neurons in the PVN and the supraoptic nucleus (SON) of the hypothalamus. It plays a key role in facilitating childbirth and lactation, as well as exhibiting anxiolytic and analgesic effects [42,43]. In healthy individuals, oxytocin regulates fear and anxiety responses by reducing amygdala activity while simultaneously enhancing the regulatory function of the prefrontal cortex [44]. However, research conducted by Zhuo’s team showed that oxytocin functionality is upregulated following chronic pain, selectively alleviating anxiety-related behaviors and presynaptic long-term potentiation associated with anxiety caused by chronic pain. This effect is likely mediated by the excitation of inhibitory neurons in the cerebral cortex [28]. Liu et al. observed that daily intranasal administration of oxytocin (2.4 μg for 28 days) helped mitigate depressive-like behaviors, though it did not affect mechanical allodynia. Oxytocin delivered intranasally suppressed the activation of both microglia and astrocytes, while simultaneously increasing the expression of oxytocin receptors that were previously downregulated [45]. Therefore, we hypothesize that prolonged chronic pain comorbidity may lead to oxytocin receptor downregulation and functional impairment, thereby increasing sensitivity to pain and negative emotions. Additionally, in a healthy state, oxytocin interacts synergistically with the serotonin and dopamine systems, facilitating emotional reward and the maintenance of close relationships. However, in the context of pain–depression comorbidity, the function of monoaminergic neurotransmitter systems may be impaired (e.g., decreased 5-HT levels or reduced receptor sensitivity), thereby weakening the regulatory effects of oxytocin. This diminished synergy may manifest as reduced emotional buffering capacity and heightened tendencies toward anxiety or depression [46,47]. Nevertheless, intranasal oxytocin holds the potential for alleviating depressive symptoms in patients with neuropathic pain, warranting further investigation.

### 2.2. Functional Remodeling of Neural Circuits

**Figure 1:**
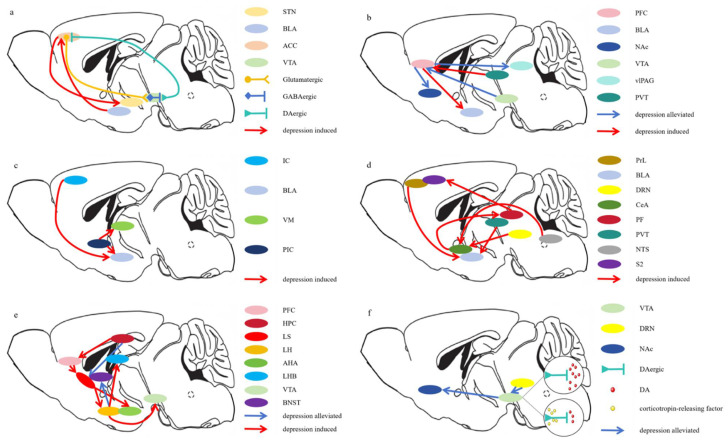
Neural Circuits. (**a**) Anterior cingulate cortex and its related circuits. (**b**) Prefrontal cortex and its related circuits. (**c**) Insular cortex and its related circuits. (**d**) Amygdala-related circuits. (**e**) Hypothalamic circuits, hippocampal circuits, and lateral septal nucleus and their circuits. (**f**) VTA and its related circuits. STN: subthalamic nucleus; BLA: basolateral amygdala; ACC: anterior cingulate cortex; VTA: ventral tegmental area; mPFC: medical prefrontal cortex; NAc: nucleus accumbens; vlPAG: ventrolateral periaqueductal gray; PVT: paraventricular thalamus; IC: insular cortex; VM: ventromedial thalamus; PrL: prelimbic cortex; DRN: dorsal raphe nucleus; CeA: central amygdala; PF: parafascicular nucleus; NTS: nucleus of the solitary tract; S2: secondary somatosensory cortex; HPC: hippocampus; LS: lateral septum; LH: lateral hypothalamus; AHA: anteromedial hypothalamic area; LHb: lateral habenula; BNST: bed nucleus of the stria terminalis; DA: dopamine.

In imaging studies of acute pain in human subjects, the most frequently activated regions include the primary somatosensory cortex (S1), secondary somatosensory cortex (S2), anterior cingulate cortex (ACC), insular cortex (IC), prefrontal cortex (PFC), thalamus, nucleus accumbens (NAc), and amygdala [48]. Among these, the ACC, PFC, IC, NAc, and amygdala are associated with the emotional components of pain. However, chronic pain can lead to persistent activation of the cortical-limbic circuit, inducing functional and anatomical changes within these brain regions, such as a reduction in gray matter volume observed in the ACC, PFC, and IC [49]. This remodeling results in emotional and cognitive dysfunction [50,51,52]. We primarily investigate the neural circuits and neurotransmitters in brain regions associated with chronic pain and mood disorders (Figure 1, Table 2).

#### 2.2.1. Anterior Cingulate Cortex and Its Related Circuits

The anterior cingulate cortex (ACC) plays a crucial role in emotion, autonomic regulation, pain processing, attention, memory, and decision making. Hyperactivity of the ACC is a key driver of the emotional impacts associated with neuropathic pain [74]. Electrophysiological recordings in chronic pain animal models have shown a sustained increase in excitatory synaptic transmission and a decrease in inhibitory synaptic transmission in the ACC, which confirms that extended exposure to pain leads to enduring alterations in synaptic plasticity [75]. Research has found that the hyperactivity of the ACC may be due to the activation of the TIAM1 protein in the pyramidal neurons of the ACC, which leads to an increase in spine density on the neuritic trees. This increased spine density enhances the number and strength of connections between neurons, which have been shown to elicit hypersensitivity and are associated with depression [76]. Patients with pain comorbidities commonly report chronic pain, fatigue, sleep disturbances, low mood, and physical limitations. These symptoms interact with one another, significantly affecting daily functioning and quality of life [77]. Functional magnetic resonance imaging studies of these patients also show increased activity in the ACC [48]. In healthy controls, pain-related modulation occurs across a broad voxel distribution within the ACC. Conversely, in chronic pain patients, this modulation is confined to a localized focal region of the ACC [78].

Wang et al. first discovered an important positive feedback neural circuit between the pain processing center (ACC) and the emotional center (ventral tegmental area, VTA). This circuit is composed of ACC glutamatergic neurons (ACC^Glu^), VTA intermediate GABAergic neurons (VTA^GABA^), and VTA dopaminergic neurons (VTA^DA^) connected in sequence. ACC^Glu^ neurons indirectly inhibit VTA^DA^ neurons via VTA^GABA^ neurons, thereby mediating the negative emotional changes associated with chronic pain. Simultaneously, VTA^DA^ neurons project back to ACC^Glu^ neurons to mediate the feedback regulation of negative emotions on pain abnormalities. Thus, the ACC^Glu^–VTA^GABA^–VTA^DA^–ACC^Glu^ circuit serves as a positive feedback neural circuit between the pain processing center and the emotional center, functioning as a core mechanism for the interaction between pain perception and emotional states, as well as an essential central mechanism for the long-term maintenance and progression of chronic pain [53]. Additionally, the ACC is closely connected to other brain regions that regulate emotional states, such as the amygdala [75]. Becker et al. found that optogenetic activation of the BLA–ACC pathway can induce depressive-like behavior in naïve mice, while optogenetic inhibition of this pathway can alleviate depressive-like behavior induced by neuropathic pain [54]. Similarly, research has shown that optogenetic activation of the ACC-STN (subthalamic nucleus) neural circuit can induce bilateral hypersensitivity and depressive-like behavior in naïve mice, while the inhibition of this pathway sufficiently reduces hypersensitivity and depressive-like behavior in both SNI mice and naïve mice following STN neuronal stimulation [55]. In conclusion, the ACC is not only a critical brain region for regulating pain and emotion but also a deeper understanding of its functions and neural mechanisms is essential for developing novel therapeutic approaches targeting pain and emotional disorders. Future research should further explore the specific mechanisms of the ACC in these processes and how to improve treatment strategies for pain and emotional disorders through the modulation of these pathways.

#### 2.2.2. Prefrontal Cortex and Its Related Circuits

The prefrontal cortex plays a central role in higher cognitive functions, decision making, emotional regulation, and self-control. Notably, in contrast to the hyperactivity seen in the ACC of individuals with chronic pain, research on both humans and animals has demonstrated that chronic pain is associated with a decrease in gray matter volume and the excitability of mPFC neurons, accompanied by increased GABAergic tone and reduced glutamatergic currents [49,79,80,81,82,83,84]. The excitability of the mPFC is determined by both its intrinsic properties and the balance of excitatory and inhibitory inputs. Key external sources of excitatory input include the thalamus, hippocampus, and amygdala, enabling the mPFC to process multiple streams of information essential for the cognitive regulation of pain. This integration supports the processing of sensory information, contextual details, and emotional elements [85]. Under physiological conditions, the mPFC exerts top-down inhibitory regulation on the activity of the amygdala. However, in adverse environments such as prolonged stress, the control exerted by the mPFC diminishes, leading to abnormal activation of the amygdala and subsequent emotional and behavioral disturbances. Pan et al. discovered that chronic stress results in an imbalance between excitatory and inhibitory neurons in the dorsomedial prefrontal cortex and the basolateral amygdala (BLA) [86].

Bagot et al. found that optogenetic activation of the glutamatergic neurons from the mPFC to the NAc can alleviate depression induced by chronic stress [56]. Chaudhury et al. reported that optogenetic inhibition of the neural circuit from the VTA to the mPFC exacerbates depression resulting from chronic stress [57]. Cai et al. demonstrated that optogenetic activation of the dorsomedial prefrontal cortex to the BLA pathway triggers a negative emotional behavioral state, increasing behaviors associated with anxiety and depression, along with aversive place avoidance. In contrast, inhibiting this pathway reversed these effects, decreasing anxiety and depression-like behaviors while enhancing preference for rewarding environments [87]. Li et al. found that chronic pain significantly reduces the activity of projection neurons in the dmPFC→vlPAG pathway, and optogenetic activation of the dmPFC→vlPAG neural pathway exhibits analgesic and anxiolytic effects in a chronic pain model (common peroneal nerve ligation) [58]. Luo et al. showed that chronic pain activates excitatory neurons in the posterior paraventricular thalamus (pPVT), which project to the mPFC, activating nNOS+ neurons in the mPFC and resulting in increased nitric oxide production. The abnormally elevated nitric oxide (NO) leads to the nitrosylation of AMPA receptors, facilitating their translocation to the cell membrane and ultimately enhancing synaptic transmission in CaMKIIα neurons within the mPFC, thereby mediating anxiety behavior induced by chronic pain [59]. This complex regulatory mechanism reveals how the mPFC participates in the modulation of pain and emotion through various pathways, providing a scientific basis for the development of new therapeutic approaches targeting these pathways.

#### 2.2.3. Insular Cortex and Its Related Circuits

The insular cortex is a key region for processing sensory information from the body, particularly related to pain perception [88,89]. A neuroimaging meta-analysis found that, in response to pain stimuli in healthy individuals, the dorsal insula is repeatedly activated, with multiple independent pain-related areas distributed along its boundaries, the most closely associated with pain being the dorsal and posterior insula. Additionally, in patients with depression, the emotional peak also shifts to the dorsal insula, which is the region related to bodily pain in healthy individuals [90], indicating that the insular cortex plays a central role in integrating pain and emotional information. Marian et al. conducted diffusion tensor imaging on patients with chronic migraine and identified abnormalities in the anterior white matter tracts of the anterior insula, particularly in the right hemisphere, which are associated with pain modulation, emotion, and cognition [91].

Research has shown that the optogenetic activation of glutamatergic neurons in the insular cortex (IC^Glu^) projecting to the basolateral amygdala (BLA^Glu^) can modulate the sensory and emotional components of pain [60]. Patients with depression often exhibit reduced motivation; however, a study by Deng et al. found that activating deep-layer (layer 5B) neurons in the insular cortex to the nucleus of the solitary tract enhances motivation in mice, potentially providing new insights and directions for the treatment of related psychiatric disorders such as depression [92]. Chen et al. demonstrated that in SNI mice, there is a notable increase in monosynaptic glutamatergic projections from the posterior insular cortex to the BLA and ventromedial thalamus (VM), coupled with heightened neuronal activity in these regions. Inhibition of the PIC→BLA and PIC→VM projections alleviates pain and exhibits antidepressant-like effects [61]. Thus, research on the insular cortex not only aids in a better understanding of the biological basis of pain and emotional disorders but may also guide the development of new therapeutic strategies. Future studies may further explore how modulating the activity of the insular cortex can treat complex pain comorbidities, opening new avenues for clinical applications.

#### 2.2.4. Amygdala and Its Related Circuits

The amygdala plays a crucial role in emotional processing, particularly in the context of fear and anxiety. In pain-related emotional disorders, the amygdala may become particularly active due to its involvement in emotional responses, thereby exacerbating pain perception and showing a positive correlation with depression scores [93]. The strengthened functional connectivity between the amygdala and the prefrontal cortex may result in the impaired regulation of negative emotions associated with pain [94]. Additionally, the fatigue and sleep disturbances reported by patients with comorbid pain may be related to excessive activation of the amygdala [95].

Gao et al. found that the prelimbic to BLA pathway mediates anxiety induced by chronic pain, and the optogenetic inhibition of this pathway reversed anxiety-like behaviors in mice [62]. The dorsal raphe nucleus (DRN) serotonin neurons project to the CeA, where they express somatostatin (CeA^SOM^) as well as non-somatostatin-expressing interneurons. CeA^SOM^ neurons directly project to the lateral habenula (LHb), a region known to be associated with depression; optogenetic inhibition of the DRN 5-HT to CeA^SOM^ pathway can alleviate depression-like behaviors in male mice with chronic pain models [16]. Tang et al. discovered that increased excitability of glutamatergic neurons in the PVT led to enhanced excitatory input to BLA neurons, and the optogenetic activation or inhibition of the PVT–BLA pathway could bidirectionally modulate pain-related behaviors and anxiety-like phenotypes [63]. Zhu et al. reported that GABAergic neurons from the central nucleus of the amygdala (CeA^GABA^) project to glutamatergic neurons in the perifornical area (PF^Glu^), which directly transmit signals to neurons in the secondary somatosensory cortex (S2). In mice with pain-related emotional disorders, enhanced inhibition of the CeA^GABA^ to PF^Glu^ to S2 pathway was observed, and reversing this pathway using chemical or optogenetic methods alleviated chronic pain symptoms [64]. He et al. found that the circuit from glutamatergic neurons in the nucleus of the solitary tract (NTS^Glu^) to somatostatin-expressing neurons in the central nucleus of the amygdala (CeA^SOM^) mediates depression-like behaviors. The inhibition or ablation of NTS^Glu^ neurons could alleviate these mice’s depressive behaviors but did not reduce hypersensitivity [65]. In summary, the amygdala serves as a key brain region in processing pain and emotional disorders. Through its diverse neural connections and neurotransmission roles, it is crucial in orchestrating the regulation of these intricate physiological processes. In-depth research on the amygdala not only aids in understanding its functions in neurophysiology and behavioral phenotypes but may also provide a scientific basis for developing novel therapeutic approaches targeting pain and emotional disorders.

#### 2.2.5. Hypothalamic and Its Related Circuits

The hypothalamus plays a crucial role in regulating fundamental bodily functions, particularly in the management of stress and pain responses. By influencing the endocrine system and the autonomic nervous system, the hypothalamus can modulate complex physiological and emotional responses. Blackburn’s research indicates that dysfunction of the HPA axis is a pathogenic factor in chronic pain and depression [96]. Oxytocin in the PVN–PFC circuit selectively enhances PFC population activity in response to nociceptive input. Activation of the PFC by oxytocin or optogenetic stimulation of oxytocinergic PVN projections alleviates pain, including its affective component [66]. Li et al. found that inflammatory pain also reduces the activity of CeA neurons. Optogenetic activation of the PVN^oxytocin^-CeA circuit can prevent anxiety-like behavior in the inflammatory pain response [67]. Iwasaki et al. demonstrated that in both female and male rats, optogenetic activation of parvocellular oxytocinergic neuronal axons releasing oxytocin in the vlPAG or chemogenetic activation of GABAergic neurons expressing oxytocin receptors in the vlPAG leads to the indirect inhibition of sensory neuronal activity in the spinal cord, thereby producing potent analgesic effects. However, this activation has no impact on the affective component of pain [68]. Gu et al. found that glutamatergic neurons in the lateral hypothalamus (LH) can project to the LHb; this excitatory LH-LHb circuit is involved in the central mechanisms of neuropathic pain, with the activation of this circuit leading to enhanced responses to mechanical and thermal stimuli [69]. Ma et al. identified a new pain-regulating neural circuit involving the LH^GABA^ to the VTA, which may promote specific dopamine release and BDNF signaling by targeting VTA GABAergic neurons [70]. Blocking the LH-to-BNST (bed nucleus of the stria terminalis) pathway leads to an increase in anxiety-like behaviors in SNI mice [71]. Furthermore, Wang et al. discovered that acute pain stimulation activates lateral septum (LS) neurons, inducing anxiety-like behaviors. The activation of GABAergic neurons projecting from the LS to the lateral hypothalamus (LH) is critical for regulating both pain and anxiety [9]. In summary, the hypothalamus provides a multilayered impact on the regulation of pain and emotional disorders through its complex neural networks and neurochemical mediators. Research on the hypothalamus offers new scientific insights and potential clinical applications in the management of pain and emotions.

#### 2.2.6. Hippocampal and Its Related Circuits

The hippocampus plays a central role in the limbic system, being critical not only for learning and memory functions but also for emotional regulation and the integration of sensory–motor information. Neuroimaging studies have shown significant structural and functional changes in the hippocampus of chronic pain patients, such as reduced hippocampal gray matter volume [97], shape alterations [98], and disrupted functional connectivity [99]. These changes are closely associated with the persistence of pain and the development of emotional disorders. According to Noorani et al., pain-driven hippocampal atrophy in trigeminal neuralgia patients contributes to increased anxiety and depression. Effective therapeutic interventions can restore hippocampal structure to normal [100]. Ploghaus similarly observed that the hippocampal entorhinal cortex responds differently to identical pain stimuli. Under anxiety, the hippocampus amplifies aversive events, promoting adaptive behaviors to anticipate worst-case scenarios [101].

Further research has revealed the role of the hippocampus in chronic pain and its comorbid emotional disorders [102,103,104]. Zhang et al. found that trigeminal neuralgia led to increased excitatory synaptic transmission from CaMKIIα neurons in the ventral hippocampus (vHPC) to inhibitory neurons in the medial prefrontal cortex (mPFC) [26]. These inhibitory neurons were linked to an elevated expression of CRH, which in turn activated CRHR1. This activation enhanced the feedforward inhibition of pyramidal neurons in layer V of the mPFC, leading to behaviors resembling anxiety and depression. The inhibition of the vHPC^CaMKIIα^-mPFC^CRH^ pathway was shown to alleviate anxiety and depression induced by trigeminal neuralgia. Currently, research on the involvement of the hippocampus in chronic pain and emotional disorders is limited, and the specific mechanisms warrant further exploration. Studies targeting the hippocampus not only enhance our understanding of the neurobiological mechanisms underlying pain and emotional disorders but may also provide theoretical foundations and potential targets for the development of novel therapeutic strategies for these conditions.

#### 2.2.7. Lateral Septal Nucleus and Its Related Circuits

The lateral septal nucleus (LS) is part of the limbic system, located between the frontal area and the striatum, with over 85% of its neurons being GABAergic [105]. It is crucial for regulating fundamental behaviors such as emotion, motivation, addiction, and sleep [106,107,108]. Some studies have also indicated a connection between LS and pain; for example, formalin-induced nociceptive behaviors resulted in a marked increase in c-Fos expression in the LS of middle-aged female rats [109]. Additionally, the chemotherapeutic agent ambrein was shown to induce pain and anxiety-like behaviors in mice by activating GABAergic neurons in the LS (LS^GABA^) [110]. Recent research suggests that the activation of LS^GABA^ neurons projecting to the LH can trigger anxiety states comorbid with chronic pain [9]. Studies have demonstrated that the optogenetic activation of CRFR2-positive neurons in the LS mediates anxiety-like behaviors in various behavioral assays, including the light–dark box, open field test (OFT), and novel object recognition tasks. Moreover, single-synaptic projections from LS CRFR2-positive GABAergic neurons to the anterior hypothalamic area promote anxiety [72]. Conversely, the activation of the neural circuit from the infralimbic medial prefrontal cortex to LS glutamatergic neurons increases anxiety-like behaviors in the OFT and elevated plus maze (EPM) tests [73]. However, electrical stimulation of the LS and chemical activation of ventral hippocampal neurons projecting to the LS can reduce fear responses and exhibit anxiolytic effects in the EPM [106,111,112,113]. We hypothesize that the bidirectional effects of the LS on anxiety may arise from differences in neuron types and the diversity of their neural circuits. The specific neural circuits and molecular mechanisms underlying these effects warrant further investigation.

#### 2.2.8. VTA and Its Related Circuits

The persistent negative impact of pain leads to common pathological symptoms such as anhedonia and depression. A hallmark of negative emotions is the decreased motivation to engage in goal-oriented tasks, which is reflected in anhedonia. Rodent models of inflammatory pain show a reduction in dopamine neuron activity in the VTA, which plays a key role in motivation regulation. Pain enhances the inhibitory input from the medial prefrontal cortex to VTA^DA^ neurons, reducing their excitability. This decline in DA neuron activity is linked to a decrease in motivation for natural rewards, which aligns with anhedonic behavior. Targeted activation of VTA^DA^ neurons is sufficient to normalize motivation and hedonic responses to these rewards [114]. Consequently, the mesolimbic reward system is integral to the processes involved in depression and pain modulation [115].

Similarly, neuropathic pain can inhibit the pathway from the DRN^VGluT3^ neurons to VTA^DA^ neurons, leading to decreased excitability of VTA^DA^ neurons. Activation of the DRN^VGluT3^→VTA^DA^ pathway can reduce neuropathic pain and associated anhedonic-like behaviors by enhancing glutamate release, which subsequently triggers dopamine release in the medial shell of the nucleus accumbens. This process exerts analgesic and anti-anhedonic effects through D2 and D1 receptor pathways, respectively [17]. Takahashi et al. found that during chronic pain, enhanced signaling of corticotropin-releasing factor within the dlBNST leads to the tonic inhibition of the mesolimbic dopaminergic system. This neuroplastic change may be related to depressive symptoms and anhedonia under chronic pain conditions [116]. Therefore, the importance of the VTA in emotional disorders induced by chronic pain cannot be overlooked. Interventions targeting the VTA dopaminergic system may represent a potential therapeutic strategy for alleviating pain-related emotional disturbances.

## 3. Neuroimmunity

Neuroimmunity refers to the interaction between the nervous system and the immune system. It encompasses not only the process of neuroinflammation but also the relationship between the central nervous system and the peripheral immune system.

**Figure 2:**
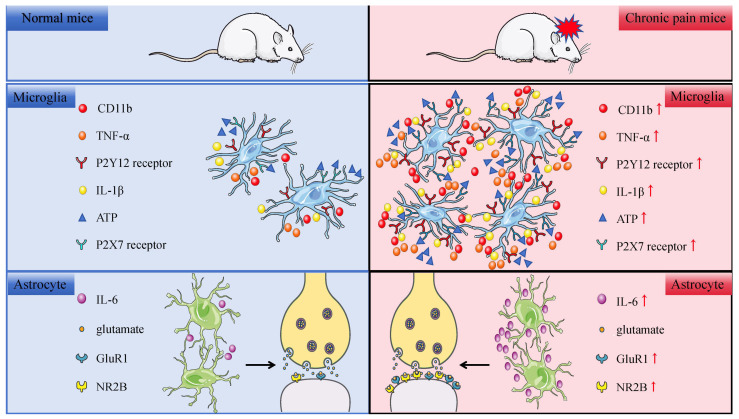
Neuroinflammation. Chronic pain can cause a significant increase in the number and volume of microglia. The transcriptional products of genes associated with microglial activation or depressive-like behaviors, such as CD11b and TNF-α, were upregulated. Activation of P2Y12 and ATP/P2X7 receptors in microglia induces IL-1β expression, leading to pain-related anxiety and depression-like behaviors. Astrocyte activation can release IL-6, enhancing the synaptic transport of GluR1 and NR2B, thereby mediating depression-like behaviors induced by nerve injury. The red upward arrow represents an increase. CD11b: cluster of differentiation 11b; TNF-α: tumor necrosis factor alpha; IL-1β: interleukin 1 Beta; ATP: adenosine triphosphate; IL-6: interleukin 6; GluR1: glutamate receptor 1; NR2B: N-methyl-D-aspartate receptor subunit 2B.

Neuroinflammation is characterized by the activation of glial cells and the release of inflammatory mediators. Glial cells play a crucial role in the onset and maintenance of central and peripheral neuropathic pain. We primarily focus on the preclinical findings regarding the activation of spinal and supraspinal microglia and astrocytes, as well as the inflammatory mediators they release, in the context of chronic pain-induced emotional disorders (Figure 2).

Barcelon et al. observed that mice developed chronic mechanical allodynia immediately following chronic constriction injury and exhibited depressive-like behaviors eight weeks after the injury. At the same time point, significant increases were noted in both the number and volume of microglia in the medial prefrontal cortex, hippocampus, and amygdala. Additionally, the expression of genes associated with microglial activation and depressive-like behaviors, such as cluster of differentiation 11b (CD11b) and tumor necrosis factor alpha (TNF-α), was notably upregulated in these regions [117]. In the ACC, microglia contribute to visceral hypersensitivity and depressive-like behaviors following colitis induced by dextran sodium sulfate. This effect is mediated through the activation of innate immune receptors TREM-1 and TREM-2 [11]. When activated, the P2Y12 receptor in microglia promotes the expression of interleukin 1 Beta (IL-1β), which is a central factor in depression. Electroacupuncture treatment significantly downregulates P2Y12 expression, attenuates microglial activation, and subsequently inhibits IL-1β expression in the mPFC, thereby alleviating visceral pain and depressive symptoms in mice with inflammatory bowel disease [118]. Taylor’s findings indicate that peripheral nerve injury activates microglia in the reward circuitry, disrupting dopaminergic signaling and reward behavior, which is also associated with emotional disturbances linked to the disruption of reward circuits [119]. Chen et al. revealed that trigeminal neuralgia activates ATP/P2X7 receptors, leading to the activation of ipsilateral microglia, which in turn drives pain-related anxiety and depressive-like behaviors through IL-1β. Developing therapies targeting microglia and the P2X7 signaling pathway may offer new treatment options for anxiety and depressive disorders related to trigeminal neuralgia [120]. Microglial activation can mediate depression and anxiety-like behaviors induced by chronic mild stress, as well as hippocampal neuroinflammatory responses [121]. Moreover, in pain-related studies, male mice cannot be used as substitutes for female mice. Agalave et al. found that the activation of the TLR4 signaling pathway induced male mouse microglia to produce more pro-nociceptive cytokines and chemokines compared to female mice [122]. Similarly, Sorge et al. demonstrated that microglia are not required for the mechanical pain hypersensitivity response in female mice; instead, female mice achieved a similar level of pain hypersensitivity through the involvement of adaptive immune cells, possibly T lymphocytes [123].

Astrocytes are essential support cells that regulate glutamate synaptic transmission. Astrocyte-driven neuroinflammation influences the equilibrium between excitation and inhibition (E/I) in the brain, and its role in comorbid pain and anxiety disrupts excitatory and inhibitory signaling in pyramidal cells of the ACC. Suppressing astrocyte activation can alleviate pain-induced anxiety and correct the E/I imbalance in the ACC [124]. Studies have shown that SNI can activate astrocytes and release IL-6 in the basolateral amygdala (BLA), enhancing the synaptic transport of glutamate receptor 1 (GluR1) and N-methyl-D-aspartate receptor subunit 2B (NR2B), thereby mediating depression-like behaviors due to nerve injury [125]. Furthermore, studies have shown that astrocyte morphology in male mice tends to be more star-shaped, while in female mice, it is more bipolar. The presence of different sex steroids influences the response of astrocytes to glutamate, which is crucial for signaling [126]. After injury, astrocyte proliferation in male rodents is more pronounced than in female mice [127].

Furthermore, the role of infiltrating immune cells, like resident microglia and astrocytes, warrants further exploration. Although the central nervous system is typically protected by the blood–brain barrier, increased permeability of the blood–brain barrier is a key feature of neuroinflammation, leading to the increased infiltration of leukocytes into the central nervous system. Studies have shown that activated immune cells, in collaboration with damaged glial cells and denervated Schwann cells, co-secrete matrix metalloproteinases, which degrade the basement membrane of endothelial cells in the blood–brain barrier, causing leakage and allowing substances that normally cannot cross a healthy blood–brain barrier to penetrate [128]. For example, tryptophan, closely associated with neurodegenerative and psychiatric disorders such as Alzheimer’s disease and depression [129], and its metabolites (such as kynurenine) can induce brain inflammation and potentially alter action potentials [130]. Recent evidence has increasingly suggested that non-neuronal cells, such as immune cells, glial cells, keratinocytes, cancer cells, and stem cells, play active roles in the onset and resolution of pain [131,132]. It is generally believed that infections caused by bacterial, fungal, and viral pathogens typically induce pain indirectly through the mediation of immune cells and the inflammatory substances they secrete. However, recent studies have shown that pathogens can also directly activate nociceptors to cause pain. For example, staphylococcus aureus produces a pore-forming toxin called α-hemolysin, which enables it to damage host cells and induce pain by directly forming pores in nociceptors. Mai et al. found that after spared nerve injury (SNI), there was a persistent increase in classical monocytes in the blood and perivascular macrophages (PVM) in several brain regions. The infiltration of monocytes into the brain may be a key factor in triggering neuropathic pain and memory deficits [133]. Kavelaars et al. found that proteinase inhibitor 16 in fibroblasts can promote the development of neuropathic pain by influencing the permeability of the blood–nerve barrier and leukocyte infiltration [134].

Currently, there is an increasing amount of research on sex differences at both the immune and neuronal levels. Studies have found that the enzyme prostaglandin D2 synthase, which enhances pain, is present at higher levels in females, and that the levels of inflammatory mediators produced by females are also higher. This not only makes them more susceptible to various autoimmune diseases but also leads to increased pain [135,136]. Analysis of neuroimmune signaling pathways in the dorsal root ganglia (DRG) of patients has revealed distinct cytokine signaling pathways in male and female neuropathic pain [137]. IL-17a-induced mechanical allodynia plays a dominant role in female mice. Low doses of IL-17a induce mechanical pain in female mice, while at higher concentrations (50 and 100 ng/mL), IL-17a increases the excitability of nociceptive receptors in the DRG neurons of male rats and enhances the sensitivity of joint nociceptors to mechanical stimuli in arthritis pain. Furthermore, IL-17a-induced pain is promoted by estrogen, while androgen suppresses it [138]. These findings provide important insights into the sex differences in pain and the role of sex hormones in pain modulation.

Therefore, understanding the changes that occur in resident immune cells and infiltrating immune cells after injury, as well as their associated effects on pain and sensitivity, may provide valuable insights for the further treatment of chronic pain and, in particular, pain-induced mood disorders. Additionally, research into the immune mechanisms of pain must consider sex as a key biological variable in order to aid in the development of effective analgesic treatments for pain conditions across all individuals.

## 4. Existing Treatment Strategies

Despite the high comorbidity of chronic pain and depression, severe depression in chronic pain patients often goes unrecognized and untreated. This may be partly due to the frequent presentation of somatic symptoms rather than emotional symptoms in these patients. Additionally, chronic pain and depression share overlapping neurobiological mechanisms. Comprehensive treatment plans for patients with both depression and pain-related somatic symptoms should include pharmacological treatments targeting serotonergic and noradrenergic neurotransmission, along with cognitive behavioral therapy, exercise, and patient education [139]. Oxytocin is known to play a role in alleviating anxiety and depressive symptoms. With the ongoing clinical research on oxytocin, it has been found to produce analgesic effects when administered intranasally, orally, or intravenously in humans. Boll et al. studied the effects of intranasal oxytocin in male patients with back pain and found that chronic low back pain patients experienced reduced heat pain sensitivity following oxytocin inhalation [140]. Tzabazis et al. provided clinical evidence for the use of intranasal oxytocin in the treatment of migraines. Their study confirmed that 24 IU of intranasal oxytocin significantly enhanced analgesic effects in chronic migraine patients who had not taken anti-inflammatory drugs within 24 h of administration [141]. Furthermore, combined analgesic therapies such as the use of analgesics, antidepressants, and psychotherapy may offer superior efficacy for treating refractory pain-related disorders.

Research has shown that acupuncture is a safe and effective treatment for depression associated with chronic pain. Compared to monotherapy, acupuncture combined with pharmacotherapy has demonstrated better outcomes [142,143]. Transcranial magnetic stimulation (TMS) can evaluate the excitability of brain circuits or the plasticity changes affecting these circuits by employing various paired-pulse paradigms. In particular, paired-pulse TMS can assess the cortical balance between inhibitory control mediated by GABAergic neurotransmission and excitatory control mediated by glutamatergic neurotransmission. Finally, repetitive TMS can activate, inhibit, or interfere with the activity of neuronal cortical networks, leading to changes in brain function that may produce lasting effects beyond the duration of the stimulation [144,145]. rTMS holds great promise in the fields of depression and chronic pain syndromes.

## 5. Future Research Directions

Current studies have primarily focused on the 5-HT and NE pathways, but recent research indicates that glutamate, GABA, CRH, and oxytocin also play significant roles in the development of pain and depression. Therefore, future research could target these pathways for the development of new pharmacological agents. Key brain regions such as the ACC, mPFC, IC, and amygdala are often implicated in the comorbidity of pain and depression. However, emerging studies have also highlighted the involvement of the hypothalamus, hippocampus, lateral septal nucleus, and ventral tegmental area in this comorbidity. Thus, investigating these additional brain regions may offer new avenues for future research on the interplay between pain and depression. Furthermore, future studies investigating neuroimmune interactions and linking immunotherapy with pain may provide significant promise for the management and eventual resolution of chronic pain and its associated emotional disorders.

## 6. Conclusions

In this review, we comprehensively summarize the intricate neurobiological mechanisms underlying emotional disorders induced by chronic pain, highlighting the significant roles of neurotransmitter dysfunction, neural circuit remodeling, and neuroimmune processes. The evidence points to a complex interplay between altered monoaminergic, GABAergic, and glutamatergic signaling, as well as structural and functional changes in key brain regions, such as the ACC, PFC, IC, and BLA. Additionally, the involvement of neuroimmune, driven by resident immune cells, infiltrating immune cells, and inflammatory mediators, underscores the bidirectional relationship between chronic pain and emotional disturbances. Despite advances in understanding these mechanisms, current treatment approaches remain suboptimal, often failing to adequately address the coexisting emotional and physical components of chronic pain. Future research should focus on developing more integrated and targeted therapeutic strategies that address the neurobiological underpinnings of this comorbidity. Such efforts are essential for improving patient outcomes, reducing the burden of chronic pain and its associated emotional disorders, and ultimately enhancing the quality of life for affected individuals.

## Figures and Tables

**Table 1:** Overview of the sources and functions of neurotransmitters.

Neurotransmitters	Source	Receptors and Main Locations	Functions	Ref
Monoaminergic neurotransmitters (5-HT, NE, DA)	5-HT:RVM NE:DLPT DA:VTA	5-HT_1_ to 5-HT_7_ receptors: cortex, hippocampus, and raphe nuclei, basal ganglia α- and β-adrenergic receptors: smooth muscles, cortex, and hippocampus, brainstem, pre-synaptic terminals D_1_-like and D_2_-like receptors: striatum, prefrontal cortex, and limbic system, basal ganglia	5-HT and NE project to the dorsal horn of the spinal cord to exert inhibitory effects on pain transmission as well as to various brain regions involved in the regulation of emotions, motor function, and cognition	[12,13,14,15,16,17]
Gamma-aminobutyric acid (GABA)	CeA, ACC	GABA_A and GABA_B receptors: cortex, hippocampus, basal ganglia, cerebellum, and spinal cord	GABA mediates inhibition of neuronal activity primarily through GABA_A and GABA_B receptors and a reduction in GABA levels may exacerbate pain and emotional symptoms	[18,19]
Glutamate (Glu)	NAc, LHb, PFC	NMDA receptors, AMPA receptors, Kainate receptors, and metabotropic glutamate receptors: cortex, hippocampus, basal ganglia, cerebellum, and spinal cord	Glutamate is involved in the neural networks of pain perception and emotional regulation and also participates in central sensitization through the activation of spinal cord neurons via AMPA and NMDA receptors	[20,21,22]
Corticotropin-releasing hormone (CRH)	PV, LC/NE, mPFC	CRH receptor 1 (CRH-R1) and CRH receptor 2 (CRH-R2): hypothalamus, cortex, amygdala, brainstem, and various peripheral tissues	CRH neurons play a role in the stress hypothesis of anxiety and depression through the hypothalamic–pituitary–adrenal axis, whose activation may indirectly reflect increased serotonergic tension in the central nervous system	[23,24,25,26,27]
Oxytocin	PVN, SON	oxytocin receptors (OTR): hypothalamus, brainstem, cortex, amygdala, uterus, mammary glands, and various other peripheral tissues	Oxytocin selectively alleviates anxiety-related behaviors and presynaptic long-term potentiation associated with anxiety caused by chronic pain	[28]

5-HT: serotonin; RVM: rostral ventromedial medulla; NE: norepinephrine; DLPT: dorsolateral pontine tegmentum; DA: dopamine; VTA: ventral tegmental area; CeA: central nucleus; ACC: anterior cingulate cortex; NAc: nucleus accumbens; LHb: lateral habenula; PFC: prefrontal cortex; PVN: paraventricular nucleus; LC/NE: locus coeruleus/norepinephrine; SON: supraoptic nucleus; AMPA: α-amino-3-hydroxy-5-methyl-4-isoxazolepropionic acid; NMDA: N-methyl-D-aspartate.

**Table 2:** Neural circuits and key neurotransmitters, receptors, and functions.

Neural Circuits	Neurotransmitters	Receptors	Neurotransmitter Functions	Key Findings	Ref
ACC^Glu^-VTA^GABA^-VTA^DA^-ACC^Glu^	DA	D2R	Excitatory	Forming a positive feedback loop that mediates the mutual promotion of chronic pain comorbidity	[53]
BLA-ACC	/	/	/	Overactive in pain comorbidity	[54]
ACC-STN	/	/	/	Overactive in chronic pain	[55]
mPFC^Glu^-NAc	Glu	/	Excitatory	Activation of the circuit alleviates depression induced by chronic stress	[56]
VTA^DA^-mPFC	/	/	/	Inhibition of this circuit can exacerbate chronic stress-induced depression	[57]
dmPFC^GABA^→vlPAG^Glu^	GABA/Glu	GABA_AR/mGluR1	Inhibitory/Excitatory	Activation of the circuit, or administration of GABA_AR antagonists and mGluR1 to the dmPFC produced analgesic and anxiolytic effects	[58]
pPVT^Glu^-mPFC^nNOS+^	NO, Glu	AMPA	Nitrosylate the AMPA receptor	pPVT excitatory neurons drive chronic pain-induced anxiety through activation of vmPFC nNOS-expressing neurons, resulting in NO-mediated AMPAR trafficking in vmPFC pyramidal neurons	[59]
IC^Glu^-BLA^Glu^	/	/	/	Overactive in chronic pain	[60]
PIC^Glu^-BLA/VM	/	/	/	Both projections were enhanced accompanied by hyperactivity of PIC, BLA, and VM neurons in SNI mice	[61]
PrL-BLA	/	/	/	Inhibition of the circuit improved anxiety but not pain sensitivity	[62]
DRN^5-HT^-CeA^SOM^-LHb	5-HT	5-HT_2A_R	Excitatory	Activation of this circuit can alleviate pain comorbid depression	[16]
PVT^Glu^-BLA	Glu	AMPA, NMDA	Excitatory	Chronic pain increases the activity of PVT^Glu^, resulting in increased excitatory inputs to BLA	[63]
CeA^GABA^-PF^Glu^-S2	GABA	GABA_AR	Inhibitory	Enhanced inhibition of the circuit was found in mice with comorbid depressive symptoms of pain	[64]
NTS^Glu^-CeA^SOM^	/	/	/	Activated in pain comorbidity	[65]
PVN^oxytocin^-PFC^Glu^	Oxytocin	OTR	Excitatory	Oxytocin in the PVN-PFC circuit alleviates pain and its affective component	[66]
PVN^oxytocin^-CeA	Oxytocin	/	/	Oxytocin has an anxiolytic effect, and activation of this circuit exerts analgesic and anxiolytic effects	[67]
PVN^oxytocin^-vlPAG^GABA^	Oxytocin	OTR	Excitatory	Activation of this circuit induces potent analgesic effects without affecting the affective component of pain	[68]
LH^Glu^-LHb	/	/	/	Overactive in chronic pain	[69]
LH^GABA^-VTA^GABA^-VTA^DA^	DA	/	/	The circuit regulated pain sensation by targeting local GABAergic interneurons to disinhibit the mesolimbic DA circuit	[70]
LH-BNST	/	/	/	Chronic pain increases inhibitory synaptic inputs and induces anxiety-like behaviors	[71]
LS^GABA^-LH	/	/	/	LS GABAergic neurons are activated in pain comorbidity with increased projections to the LH	[9]
vHPC^Glu^-mPFC^GABA^	CRH	CRHR1, GABA_AR	Inhibitory	In pain comorbidity, excitatory synaptic transmission in this circuit is enhanced, leading to feedforward inhibition of the mPFC via CRHR1	[26]
LS^Crfr2^-AHA	Corticosteroid	CRFR2	/	Activation of LS^Crfr2^ neurons can induce both behavioral and neuroendocrine dimensions of a persistent anxiety state	[72]
IL-LS	/	/	/	IL-LS projections promote anxiety-related behaviors	[73]
DRN^VGLUT3^-VTA^DA^	Glu, DA	D2R, D1R	Excitatory	Activation of this circuit releases glutamate, promoting DA release in the medial shell of the nucleus accumbens, which exerts analgesic effects via D2 receptors and counteracts anhedonia via D1 receptors	[17]

GABA: gamma-aminobutyric acid; GABA_AR: GABA A receptor; Glu: glutamate; AMPA: α-amino-3-hydroxy-5-methyl-4-isoxazolepropionic acid; NMDA: N-methyl-D-aspartate; DA: dopamine; D1R: dopamine D1 receptor; D2R: dopamine D2 receptor; 5-HT: -Hydroxytryptamine; 5-HT_2A_R: 5-HT 2A Receptor; CRH: Corticotropin-releasing hormone; CRHR1: CRH Receptor 1; CRFR2: Corticotropin-Releasing Factor Receptor 2; OTR: oxytocin receptor; NO: nitric oxide; VGLUT3: vesicular glutamate transporter 3; SOM: somatostatin; PF: perifornical area; RVM: rostral ventromedial medulla; CeA: central amygdala; ACC: anterior cingulate cortex; NAc: nucleus accumbens; LHb: lateral habenula; mPFC: medical prefrontal cortex; PVN: paraventricular nucleus; S2: secondary somatosensory cortex; IC: insular cortex; VTA: ventral tegmental area; STN: subthalamic nucleus; BLA: basolateral amygdala; pPVT: posterior paraventricular thalamus; VM: ventromedial thalamus; DRN: dorsal raphe nucleus; LH: lateral hypothalamus; BNST: bed nucleus of the stria terminalis; LS: lateral septum; vHPC: ventral hippocampus; vlPAG: ventrolateral periaqueductal gray; PrL: prelimbic cortex; NTS: nucleus of the solitary tract; AHA: anteromedial hypothalamic area; PF: parafascicular nucleus.
